# Association between maternal and perinatal outcomes and histological changes in the placenta of patients with Covid-19: A cohort study

**DOI:** 10.1097/MD.0000000000038171

**Published:** 2024-05-24

**Authors:** Luiza Rocha de Souza, Melania Maria Ramos Amorim, Alex Sandro Souza, Brena Carvalho Pinto de Melo, Christiane Tiné Cantilino, Maria Alice de Oliveira Saunders, Maria Jucá de Petribú, Luciana Soares Lúcio, Juliana Rodrigues Marinho, Maria Eduarda Virgínia de Oliveira Correia, Leila Katz

**Affiliations:** a Master’s Program of Comprehensive Health at IMIP, Recife, Brazil; b High Risk Pregnancy Unit at IMIP, Recife, Brazil; c Professor of the Postgraduate Program at IMIP, Recife, Brazil; d Department of Fetal Medicine at IMIP, Recife, Brazil; e Simulation Center at Faculdade Pernambucana de Saúde (Csim), Recife, Brazil; f Department of Pathology at iMIP, Recife, Brazil; g Faculdade Pernambucana de Saúde, Recife, Brazil; h Obstetric Intensive Care Unit at IMIP, Recife, Brazil.

**Keywords:** Covid-19, maternal death, placenta, pregnancy

## Abstract

Although studies evaluated placental involvement in Covid-19 patients, few have assessed its association with clinical repercussions. The study aimed to determine the association between the clinical status and maternal and perinatal outcomes of patients with Covid-19 at delivery and changes in placental histology. It is so far the largest cohort evaluating placentas of patients infected by the SARS-CoV-2. A secondary analysis was conducted of a database from which a cohort of 226 patients, who tested real-time polymerase chain reaction-positive for Covid-19 at delivery and whose placentas were collected and submitted to pathology, was selected for inclusion. One or more types of histological changes were detected in 44.7% of the 226 placentas evaluated. The most common abnormalities were maternal vascular malperfusion (38%), evidence of inflammation/infection (9.3%), fetal vascular malperfusion (0.8%), fibrinoid changes and intervillous thrombi (0.4%). Oxygen use (*P* = .01) and need for admission to an intensive care unit (ICU) (*P* = .04) were less common in patients with placental findings, and hospital stay was shorter in these patients (*P* = .04). There were more fetal deaths among patients with evidence of inflammation/infection (*P* = .02). Fetal death, albeit uncommon, is associated with findings of inflammation/infection. Oxygen use and need for admission to an ICU were less common among patients with placental findings, probably due to the pregnancy being interrupted early. None of the other findings was associated with maternal clinical status or with adverse perinatal outcome.

## 1. Introduction

Initially believed to be a respiratory disease, the COVID-19 is now classified as a systemic disease due to the abundance of receptors for the virus, such as the angiotensin-converting enzyme 2 and transmembrane serine protease 2, in various tissues.^[1]^

The low incidence of Covid-19 in the population of pregnant women in Asian countries led to the misconception that, unlike other respiratory viruses, the pregnant population would have a low risk for severe disease.^[1–3]^ However, as cases increased worldwide, population-based studies revealed higher rates of maternal adverse outcomes such as admission to intensive care units (ICU) and death.^[2–4]^ In addition, various studies have shown that SARS-CoV-2 infection increases the risk of adverse perinatal outcomes, possibly linked to the severity of the disease.^[5]^ Considering the role of the placenta in different conditions, the answer to some Covid-19-related outcomes may also be of placental origin.

Although controversial, angiotensin-converting enzyme 2 is believed to be richly expressed in the placenta, theoretically representing an important risk of placental involvement and of vertical transmission of SARS-CoV-2.^[6,7]^ As seen during the SARS-CoV and MERS outbreaks, apart from vertical transmission, SARS-CoV-2 is responsible for significant placental involvement and marked histopathological changes.^[8,9]^ The abnormalities found are nonspecific and unassociated with placental infection; however, it is noteworthy that vascular involvement is strongly present.^[9,10]^

Despite the number of studies focusing on the histopathology of placentas with Covid-19, little is known regarding the actual repercussions of these abnormalities or the effect of certain clinical characteristics.^[5,8,9]^ Understanding the association between maternal clinical status, perinatal outcome and placental abnormalities can improve the care and follow-up provided to pregnant women with Covid-19. This study aimed to evaluate the association of clinical status and maternal and perinatal outcomes with placental abnormalities in Covid-19 patients.

## 2. Material and methods

This study used a database from a multicenter, prospective and retrospective cohort study conducted in Brazil (ClinicalTrials registration number NCT04462367). The institute’s internal review board approved the original study protocol (CAAE 65112822.2.0000.5201) and all participating women gave their written consent. The current secondary analysis considered only data collected at the *Instituto de Medicina Integral Prof Fernando Figueira* (IMIP) in Recife, Brazil, between June 2020 and June 2022, since this was the only center at which placentas were collected and analyzed.

According to the procedures implemented in the region during the pandemic, admission to IMIP was required for pregnant women with Covid-19 for care during delivery. Similarly, pregnant women at any gestational age whose condition was sufficiently severe as to require clinical treatment were to be admitted. In other cases, even asymptomatic but Covid-19-positive patients were also treated at the unit. Conversely, if Covid-19-negative, they were referred to another hospital.

### 2.1. Characteristics of the study population

The maternal characteristics evaluated were: age, ethnicity, overweight/obesity, smoking, alcohol consumption, severe acute respiratory syndrome (SARS) (defined as flu-like signs and symptoms associated with oxygen saturation < 95% or respiratory distress or tachypnea, hypotension and worsening of the clinical conditions of the primary disease), hypertensive disorders, gestational diabetes, previous diabetes, asthma and premature labor. The outcomes evaluated consisted of individual maternal outcomes, composite maternal outcome (need for oxygen, mechanical ventilation, SARS, maternal near miss and maternal death), hypoxemia and duration of hospital stay.

Neonatal outcome was also evaluated individually and as composite neonatal outcomes 1st-minute Apgar score < 7, 5th-minute Apgar score < 7, fetal death, neonatal death before 7 days and prematurity. The newborn children were tested for Covid-19 by real-time polymerase chain reaction (RT-PCR) within 72 hours of birth.

### 2.2. Collection and analysis of placentas

The placentas of women who delivered at IMIP and who tested positive for Covid-19 by RT-PCR were routinely collected and analyzed resulting in a total of 226 patients and placentas. Following delivery, the placentas were collected and deposited individually in containers with 10% buffered formaldehyde with a volume of 6 times the amount of its volume for fixation and preservation. In the pathology laboratory, the placentas were processed for microscopic evaluation. After macroscopic evaluation of the placenta, they were cleaved into fragments 3 to 5 mm thick, which were then subjected to dehydration with ethyl alcohol, clarification to remove the alcohol and infiltration with liquid paraffin. The paraffin block was subjected to microtomy to obtain ultra-thin and uniform slices and subsequent staining with hematoxylin and eosin.^[11,12]^ The sections obtained were sealed with xylene and slides were prepared and analyzed by a single experienced pathologist. Placental findings were described in accordance with the 2016 Amsterdam Placental Workshop Group Consensus Statement,^[13]^ following the framework for placental pathology reporting established by Benton et al.^[14]^

Maternal vascular malperfusion (MVM), fetal vascular malperfusion (FVM) and inflammation/infection were the parameters selected to evaluate the association between specific histological changes and maternal and perinatal outcome. These placental findings were chosen because MVM and infection were the most common abnormalities found in this study population and, in some studies, FVM is a common finding.

### 2.3. Statistical analysis

Statistical analysis was conducted using Epi-Info, version 7.2.5 (Atlanta). To describe the numerical variables, measures of central tendency and dispersion were used. Frequency distribution tables were constructed to describe the categorical variables. The chi-square test was used to evaluate the association between categorical variables, with Fisher’s exact test being used whenever pertinent. All *P* values were two-tailed. The significance level adopted was 5%.

## 3. Results

**Figure 1.**
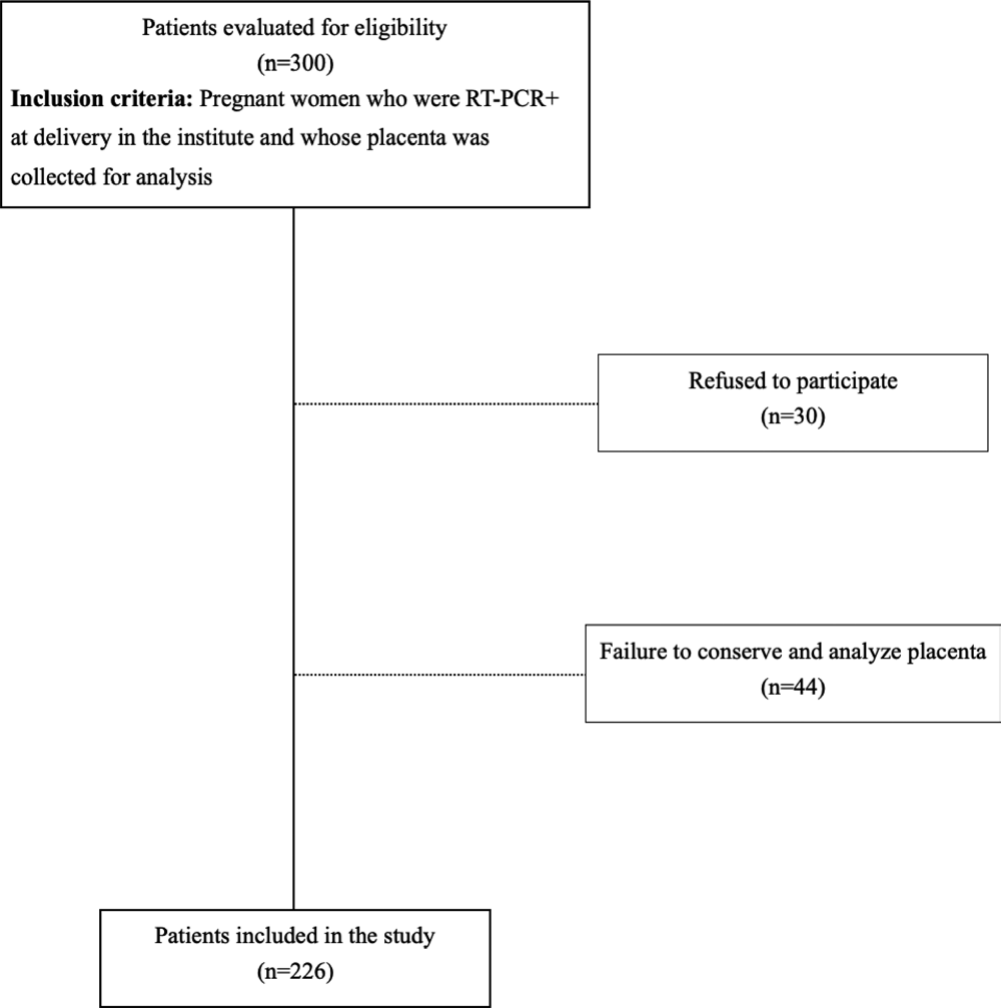
Flowchart of admission to the study.

Three hundred patients who met the eligibility criteria were approached during the placenta collection period. After eliminating the patients who refused to participate as well as cases of poorly conserved placentas, 226 RT-PCR-positive patients, who gave birth at the institute and whose placentas were collected and analyzed, were included in the study (Fig. 1).

Of 226 placentas, 101 (44.7%) had at least one histopathological change, with MVM being the most common (Table 1). The maternal characteristics can be found in the Table 2. No statistically significant difference was found between women with or without placental abnormalities.

**Table 1:** Histopathological changes in the placenta of patients with Covid-19.

Placental histological changes	n	%
Evidence of maternal vascular malperfusion (MVM)	86	38
Evidence of inflammation/infection	21	9.3
Evidence of fetal vascular malperfusion (FVM)	2	0.8
Abnormal implantation site	0	0
Fibrinoid changes and intervillous thrombi	1	0.4
Evidence of abnormal development of the placental villous tree	0	0
Evidence of uteroplacental separation	0	0
Any other histological change*	101	44.7

* Some placentas had more than one specific abnormality.

**Table 2:** Association between maternal characteristics and any histological change in the placenta of patients with Covid-19.

Maternal characteristics	Histopathological changes n = 101	No histopathological changes n = 125	*P* value
Age, years (mean/SD)	27.5/6.8	27.1/6.7	.65
Gestational age (median/IQR)	37/35–39	38/34–39	.9¶
	**n/%**	**n/%**	
Black*	9/11.4	10/10.1	.78
Overweight/obese†	15/26.3	18/27.3	.9
Smoking‡	4/6.1	2/2.9	.43∥
Alcohol use§	3/4.7	6/8.8	.49∥
SARS	31/30.7	52/41.6	.09
Hypertensive disorders	36/35.6	44/35.2	.94
Clinical diabetes	3/3.3	9/7.9	.23∥
Gestational diabetes	9/8.9	18/14.4	.10
Asthma	4/3.96	4/3.2	1.00∥
Premature labor	7/6.9	6/4.8	.57

IQR = interquartile range, SARS = severe acute respiratory syndrome, SD = standard deviation.

Data available in the medical charts for:

* 178 cases;

† 125 cases;

‡ 133 cases;

§ 130 cases.

∥ Fisher’s exact test.

¶ Mann–Whitney *U* test.

In patients with SARS, gestational age at pregnancy interruption was lower, with a median of 35 completed weeks compared to a median of 38 completed weeks for those without SARS (*P* < .001) (data not shown as table).

Regarding individual maternal outcomes, 9 maternal deaths occurred (3.9%), with more deaths among the patients with a placental abnormality although it was not statistically significant (Table 3). Thirty-one patients with a histological change (30.7%) and 52 patients with none (41.6%) had SARS, with no statistically significant difference between the groups. Conversely, the need for oxygen was associated with an absence of placental abnormalities (27.7% vs 44.0%, *P* = .01); however, when only the use of assisted mechanical ventilation (AMV) and the occurrence of hypoxia were evaluated, there was no association either with the presence or absence of placental abnormalities or with any specific abnormality (Table 3).

**Table 3:** Association between maternal outcomes and histological changes in the placenta of patients with Covid-19.

Maternal Outcome	Histological changes in general			MVM			FVM			Inflammation/infection		
	Yes (n/%) n = 101	No (n/%) n = 125	*P* value	Yes (n/%) n = 86	No (n/%) n = 140	*P* value	Yes (n/%) n = 2	No (n/%) n = 224	*P* value	Yes (n/%) n = 21	No (n/%) n = 205	*P* value
Maternal death	7/6.9	2/1.6	.08†	6/7.2	2/2.1	.06†	0/0	9/4.0	1.00†	1/4.7	8/3.9	.60†
Near miss	10/9.9	16/12.8	.50	9/10.5	17/12.1	.70	0/0	26/11.6	.60†	2/9.5	24/11.7	.76†
SARS	31/30.7	52/41.6	.09	28/32.6	55/39.3	.30	0/0	83/37.1	.28†	6/28.6	77/37.6	.41
Use of oxygen	28/27.7	55/44.0	.01	25/29.1	58/41.4	.06	0/0	83/37.0	.28	6/28.6	77/37.6	.41
AMV	11/10.9	18/14.4	.40	10/11.6	19/13.6	.60	0/0	29/12.9	.58†	3/14.3	26/12.7	.73†
Admitted to ICU	25/24.7	47/37.6	.04	22/25.6	50/35.7	.10	1/50.0	71/31.7	.58†	6/28.6	66/32.2	.73
Composite maternal outcome	38/37.6	61/48.8	.09	34/39.5	65/46.4	.31	1/50.0	98/43.7	.85†	8/38.1	91/44.4	.58
Days of hospital stay (median/IQR)	4 (3–7)	5 (2.5–10.5)	.04‡	4 (3–7)	4.5 (3–10)	.20‡	9 (1–17)	4 (3–8)	.88‡	3 (3–6)	4 (3–8)	.57‡
Respiratory symptoms	82/82.8	98/80.1	.60	69/82.1	111/81.6	.86	1/50.0	179/82.1	.30†	18/85.7	162/80.6	.46
Hypoxemia*	15/38.5	22/41.5	.76	12/38.7	25/41	.83	0/0	37/40.0	1.00†	3/50.0	34/40.0	1.00†

AMV = assisted mechanical ventilation, FVM = fetal vascular malperfusion, ICU = intensive care unit, IQR = interquartile range, MVM = maternal vascular malperfusion, SARS = severe acute respiratory syndrome.

* Blood gas analysis was only performed in 93 patients.

† Fisher’s exact test

‡ Mann–Whitney *U* test.

Composite maternal outcome was more common in patients with no histological changes (37.6% vs 48.8%); however, this difference was not statistically significant. There was no difference in the presence of respiratory symptoms between the group with any histological abnormality compared to the group with no abnormality as well as the presence of respiratory symptoms (Table 3).

Hospital stay was significantly shorter for the patients with a histological change: 4 (IQR 3–7) versus 5 (IQR 2.5–10.5), *P* = .047; however, there was no association with any specific type of abnormality. Likewise, patients admitted to the ICU were less likely to have a placental abnormality than those who did not require intensive care (24.7% vs 48.8%, *P* = .04). No association was found between admission to the ICU and any specific abnormality (Table 3).

Eight newborn infants (11.9%) were RT-PCR positive for Covid-19 in the first 72 hours of life, with no statistically significant difference between the groups. Fetal death was more common in the group with placental abnormalities (5.9% vs 1.6%); however, this difference was not statistically significant (*P* = .08). Nevertheless, when fetal death was compared between patients with and without evidence of infection/inflammation, this outcome was significantly more frequent when evidence of these abnormalities was present (14.3% vs 2.4%; *P* = .02). Unlike fetal death, no association was found between placental abnormalities in general or any specific abnormality and neonatal death prior to 7 days of life (Table 4).

**Table 4:** Association between perinatal outcomes and histological changes in the placenta of patients with Covid-19.

Perinatal Outcome	Histological changes in general			MVM			FVM	Inflammation/infection				
	Yes (n/%)	No (n/%)	*P* value	Yes (n/%)	No (n/%)	*P* value	Yes (n/%)	No (n/%)	*P* value	Yes (n/%)	No (n/%)	*P* value
RT-PCR positive*	4/13.3	4/11.7	.73	4/16.6	4/10.2	.46§	0/0	8/12.7	1.00§	0/0	8/14.0	1.00§
1st minute Apgar < 7†	15/16.8	15/12.6	.38	15/19.4	15/11.4	.11	0/0	30/14.4	1.00§	1/6.0	29/15.2	.47§
5th minute Apgar < 7‡	2/2.7	3/3.7	1.00§	2/3.0	3/2.8	1.00§	0/0	5/2.2	1.00§	1/7.1§	4/2.5	.35§
Fetal death	6/5.9§	2/1.6	.07	5/5.8§	3/2.1	.06§	1/50.0	7/3.1	.07§	3/14.3	5/2.4	.02§
Neonatal death < 7 days	2/2.0	3/2.4	1.00§	2/2.3§	3/2.1§	1.00§	0/0§	5/2.2	1.00§	0/0§	5/2.2	1.00§
Prematurity	34/33.6	48/38.4	.46	30/34.8	52/37.1	.77	1/50.0§	81/36.2	1.00§	7/33.3	75/36.6	.76
Composite perinatal outcome	44/43.6	52/41.6	.76	39/45.4	57/40.7	.50	1/50.0§	95/42	1.00§	9/42.8	87/42.4	.97

FVM = fetal vascular malperfusion, MVM = maternal vascular malperfusion.

Data available in the medical charts for:

* 63 fetuses;

† 208 patients;

‡ 171 patients.

§ Fisher’s exact test.

Eighty-two infants (36.2%) were born prematurely in this population. The frequency of prematurity was similar in the groups. Similar results were found when 1st and 5th minute Apgar scores < 7 were evaluated as a function of the presence of a histological change or the presence of any specific change (Table 4).

Composite perinatal outcome was present in 96 cases, with some form of histological change being present in 44 and absent in 52. The most common abnormalities found were: MVM (59 cases), followed by inflammation (9) and FVM (1); however, no association was found between composite outcome and any one of these findings (Table 4).

## 4. Discussion

The results of this study show that placental histological changes are common in patients with SARS-CoV2, with MVM being the most common of these changes; however, neither this abnormality nor any of the others is associated with clinical status or maternal outcome. Nevertheless, an association was found between fetal death and placental inflammation/infection. Notwithstanding, histological changes were found to be less common in patients requiring ventilatory support or admission to an ICU. Furthermore, hospital stay was shorter in patients with placental histological changes.

Shanes et al and Taglauer et al were among the first to compare placentas from women who had Covid-19 in the third trimester of pregnancy with controls.^[15,16]^ In agreement with the results of the present study, MVM was the most common abnormality in patients with Covid-19. Conversely, some studies have reported FVM as the most common abnormality.^[8,17]^

The thrombotic nature of Covid-19 has been well established and may explain the finding of MVM and FVM as the most common abnormalities.^[8,18,19]^ Diseases such as preeclampsia and factors such as smoking could be associated with this and other placental abnormalities; nevertheless, in the present study, the population was divided into 2 groups as a function of the presence or absence of histological changes, with results showing that these comorbidities did not affect our findings.^[11]^

Hypoxic-ischemic insults to the fetus and placenta can also result in placental injury, particularly MVM.^[8,19,20]^ Although no association was found here between maternal hypoxia and placental lesion, only hypoxia occurring during hospitalization was taken into account and blood gas analysis was only conducted in 93 of the 226 patients, since this test was indicated when criteria for SARS were present. The relatively few tests performed could have compromised the evaluation of a possible association through a type II error.

It would be expected that more severe patients and those with poorer outcomes would be more likely to have more placental abnormalities, since the disease severity (in addition to vertical transmission) appears to be associated with the degree of maternal viremia.^[21,22]^ While some studies have supported that hypothesis, others showed that, despite the finding of MVM in the placentas of infected patients, there were no differences between those patients and the controls.^[23,24]^ Likewise, other studies have also found no difference in placental findings between asymptomatic and symptomatic patients or between those with mild and severe disease.^[25,26]^ This finding could be explained by the fluctuating course of the disease as a result of treatment provided or by the early interruption of the pregnancy.^[17,24]^

The results of the present study differ from those of an Italian study that reported an association between ICU admission and placental histological changes, particularly MVM.^[24]^ The present sample consisted of patients with Covid-19 admitted to hospital to interrupt their pregnancy (either because they were in labor and had COVID-19 or because they had COVID-19 and a comorbidity for which interruption was indicated) or patients admitted at any gestational age whose condition was sufficiently severe as to require hospitalization. According to the protocol, patients over 34 weeks of gestational age with SARS had their pregnancy interrupted promptly in order to improve their respiratory status, with the patients then being admitted to the ICU.^[13]^

The earlier interruption of pregnancy in this population could have resulted in these patients having less time of exposure to severe disease and, consequently, less time of viremia and inflammation that could lead to placental abnormalities. The presence of a low rate of FVM (0.8%) compared to other studies with a mean of 9% further strengthens this hypothesis, since this is associated with acute disease.^[27]^

Another way of evaluating severity in infected patients is the presence of SARS; however, its association with placental abnormalities is also controversial. Another Brazilian study compared placental findings with the severity of Covid-19 and found that only cases considered moderate were associated with placental abnormalities, probably due to the longer time of expectant management.^[25]^

Here, the population was subdivided into severe and non-severe as a function of the presence or absence of SARS, with no difference between the groups insofar as the presence of histological changes or of any specific histological change is concerned. These findings are in agreement with a US study in which the groups were similarly divided.^[26]^ An Indian study evaluated 179 Covid-19 patients and also failed to find any association between severity and placental abnormalities.^[28]^ One possible explanation for this finding and for the lesser need for ICU admission is that, when clinical condition is severe, pregnancy is interrupted promptly, leaving less time for histological changes to develop.

Maternal mortality in Covid-19 patients is low, albeit significantly higher compared to uninfected pregnant women, with a rate of around 1.3% to 2%.^[29]^ This rate varies greatly from region to region and is considerably higher in poorer countries.^[29–31]^ Of the few studies that analyzed the association between placental findings and perinatal outcomes, many failed to evaluate the association between abnormalities and maternal mortality because no such cases were recorded. In the present analysis, 9 maternal deaths occurred (4% of the population); however, there was no statistically significant association with the presence of placental abnormalities. A limitation of the present study is that the moment of death was not determined, which could have supported the hypothesis regarding the earlier interruption of pregnancy.

Here, oxygen use was associated with fewer placental abnormalities in general; however, when only the use of AMV was investigated, there was no statistically significant difference. Three factors could explain this. Firstly, this study took into consideration oxygen use and AMV at any time during hospitalization (pregnancy or postpartum). Secondly, even when oxygen was required for patients who were still pregnant, as speculated by some authors, treatments could have modified the process of the disease and lesion^[17,24]^; thirdly, the criteria of intubation changed during the pandemic with early intubation being advised in the beginning.^[32]^ Pregnant women who required AMV probably had their pregnancy interrupted even earlier. On the other hand, the lower frequency of changes in patients with longer hospitalizations could also be justified. Another possible hypothesis for these and other findings in this study is that since the study analyzed placentas, the fetal component would tend to involve more consequences than the maternal component.

A large meta-analysis found that infants born to mothers infected with SARS-CoV-2 are more likely to be premature, to be admitted to an ICU, and to suffer intrauterine or perinatal death.^[33]^ In the present study, an attempt was made to identify possible causes of the worse outcome by evaluating their associations with abnormalities at placental histology.

An Indian study found that the presence of abnormalities in placental histology increased the risk of intrauterine death and 1st and 5th minute Apgar scores < 7.^[28]^ That study associated abnormal Apgar scores with the presence of retroplacental blood clots/hemorrhage and intervillous fibrin deposition but failed to find an association between intrauterine death and any specific findings.

As in the aforementioned study, the present sample was divided according to the presence or absence of specific abnormalities or of any abnormality at all. Unlike the Indian study, we failed to find any association between placental abnormalities and Apgar scores.^[28]^ Two factors could explain this. Firstly, the Indian study included placentas from 20 weeks of pregnancy onwards, possibly reflecting chronic placental lesions that would have a greater repercussion at birth.^[28]^ Conversely, the present study analyzed only placentas in the third trimester of pregnancy when they are much better developed. Furthermore, acute infection was present. Secondly, there was only one case of intervillous fibrin deposition (fibrinoid changes) and no cases of retroplacental blood clot/hemorrhage.

To evaluate intrauterine death in the present study, the population was divided into those with any placental abnormality and those with no abnormality. No statistically significant differences were found. Nevertheless, when tuhe analysis was performed for specific abnormalities, the incidence of fetal death was significantly greater among patients with evidence of placental infection/inflammation.

The association between inflammation in fetal tissues and adverse outcomes is well established in the literature.^[34]^ Since Covid-19-related fetal death is uncommon, only case series are available in the literature. A Brazilian case series described 5 fetal deaths in Covid-19 patients, all with acute chorioamnionitis.^[35]^ Other case series reported that in situations in which fetal death occurs, a frequent finding is intense placental inflammation represented by chronic histiocytic intervillositis, villitis of unknown etiology and chronic deciduitis.^[36]^ In the present study, findings of inflammation were rare and consisted of nonspecific forms of maternal and fetal inflammatory (such as chorioamnionitis) which are commonly associated with ascending genital tract infections.^[34]^ As proposed by Cardenas et al, viral infections that do not result in vertical transmission tend to cause placental lesions and facilitate bacterial infection, which could explain the finding of chorioamnionitis.^[18,37]^ No cases of villitis, chronic histiocytic intervillositis or villitis of unknown etiology were found; however, the association between death and evidence of inflammation could reflect the already established association with chorioamnionitis of any other etiology.^[34]^

The possibility of vertical transmission is also a focus of attention during a pandemic involving a new virus.^[38]^ Although there were 8 RT-PCR-positive cases in less than 72 hours, few newborns were tested and RNA could not be detected in placentas. Consequently, we can neither affirm nor rule out vertical transmission, as mandated in protocols.^[39]^

The present study involves some significant strongpoints, particularly the sample size and specifically with respect to the number of placentas analyzed. Due to the scarcity of studies involving placentas in different populations, generalizations regarding the most common findings in Covid-19 patients are difficult. Therefore, it is hoped that the results of this study will contribute to future systematic reviews with meta-analyses. These data are in agreement with those of other investigators who failed to find any direct association between maternal clinical status and placental abnormalities, reflecting the probable existence of little understood mechanisms of local protection against SARS-CoV-2 and the association between low vertical transmission and frequent placental injury.

Nevertheless, there are some limitations associated with the present study; firstly, the inadequate completion of patient records, hampering access to certain important data. Furthermore, this study failed to determine the time between the first symptoms and the moment of pregnancy interruption or the exact time of maternal death (in relation to symptoms at delivery), of admission to the ICU, and of oxygen use, which could support the hypothesis of fewer findings when pregnancy is interrupted early. Although we speculate that the data of vaccination status would have revealed different findings, our sample had a very small number of vaccinated patients to allow assessment of this potential impact, as the study was carried out at the height of the pandemic, when vaccination of the obstetric population in the country had not yet begun. Due to the lack of financial resources, the SARS-CoV could not be tested in the placental tissues which could have corroborated the possibility of infection of the placental tissue leading to the histologic consequences. Finally, due to the hospital admission flow at this institute, only patients currently having Covid-19 could be analyzed. Those who had had Covid-19 earlier but who did not have the active disease at delivery were not included; however, placental findings could have been present and could have affected outcomes.

Although difficult (due to a better control and vaccination against COVID-19), we recommend for future research a new assessment of placental findings and maternal and neonatal outcomes, specifying the moment of birth, the beginning of each therapy and the moment of maternal death, situations that were limiting in our study. We also believe it is important to include the variable “vaccination status” in future studies to assess whether prior and adequate maternal vaccination against COVID-19 can modify the magnitude of placental changes.

In conclusion, placental abnormalities are common, with the most frequent abnormality being MVM. Abnormalities, however, are not associated either with maternal clinical status or with most adverse perinatal outcomes except for fetal death, which, albeit uncommon, was associated with evidence of inflammation/infection. The use of oxygen therapy and admission to an ICU were factors associated with fewer placental abnormalities, probably due to the earlier interruption of pregnancy.

## Author contributions

**Conceptualization:** Luiza Rocha de Souza, Alex Sandro Souza, Leila Katz, Melania Maria Amorim.

**Data curation:** Luiza Rocha de Souza, Leila Katz.

**Formal analysis:** Luiza Rocha de Souza, Leila Katz.

**Investigation:** Luiza Rocha de Souza, Christiane Tiné Cantilino, Maria Alice de Oliveira Saunders, Maria Jucá de Petribú, Luciana Soares Lúcio, Juliana Rodrigues Marinho, Maria Eduarda Virgínia de Oliveira Correia.

**Methodology:** Luiza Rocha de Souza, Leila Katz, Melania Maria Ramos Amorim.

**Project administration:** Leila Katz.

**Supervision:** Leila Katz, Brena Carvalho Pinto de Melo, Melania Maria Ramos Amorim.

**Validation:** Christiane Tiné Cantilino, Melania Maria Ramos Amorim.

**Writing – original draft:** Luiza Rocha de Souza.

**Writing – review & editing:** Luiza Rocha de Souza, Leila Katz.
